# Supplementary figures and images for: Genome-scale investigation of phenotypically distinct but nearly clonal Trichoderma strains
Source: PeerJ. 2016 May 12;4:e2023. doi: 10.7717/peerj.2023 (PMC4868595; doi:10.7717/peerj.2023)

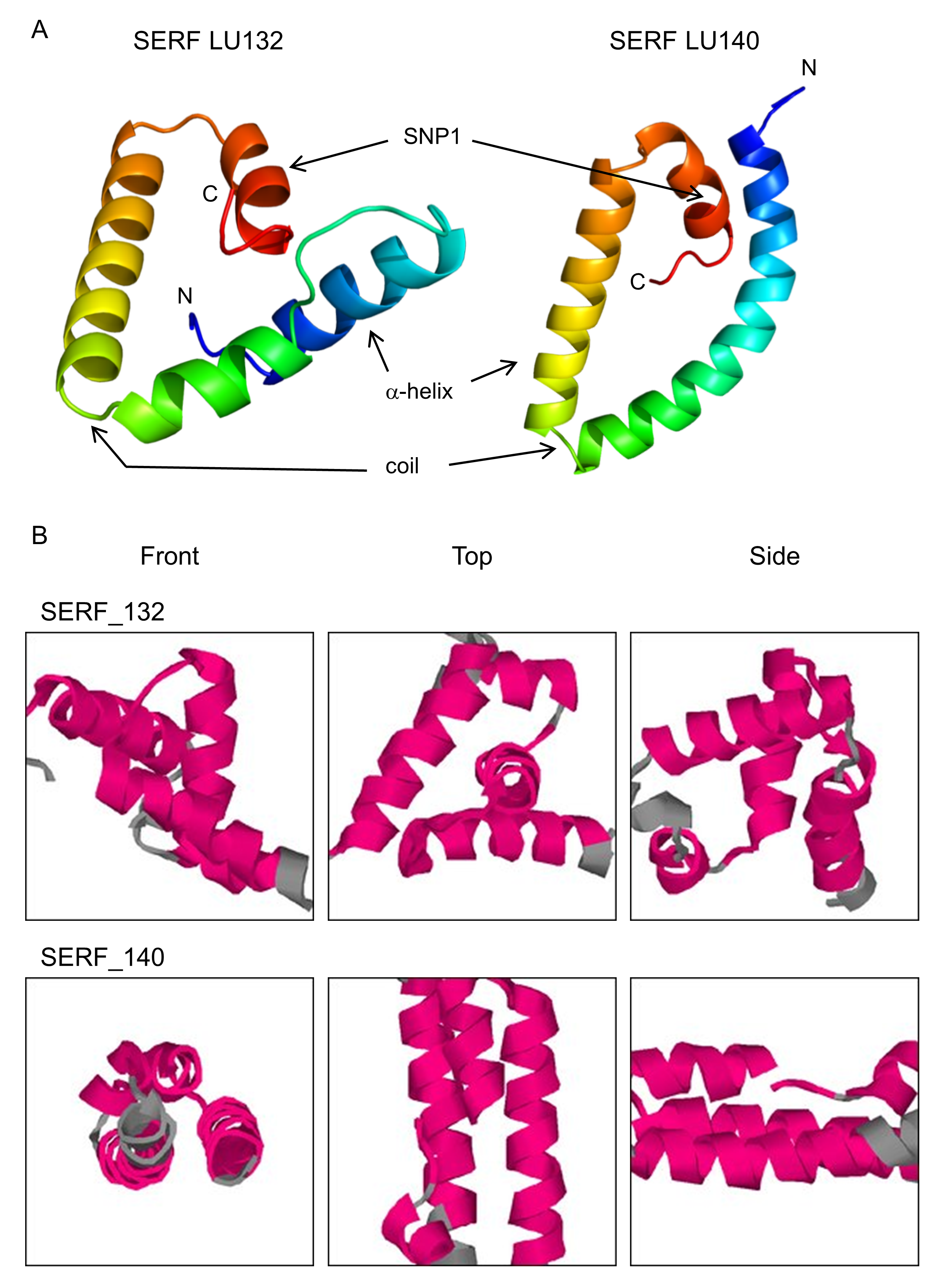

Supplement: Figure S1 — (A) Phyre2 prediction, blue, N terminus; red, C terminus; four α-helices for LU132 and three for LU140. The approximate location of the amino acid change is indicated by “SNP1”. (B) Sam-T08 prediction, red for α-helix and grey for other, four α-helices for LU132 and three for LU140. [file peerj-04-2023-s001.png]

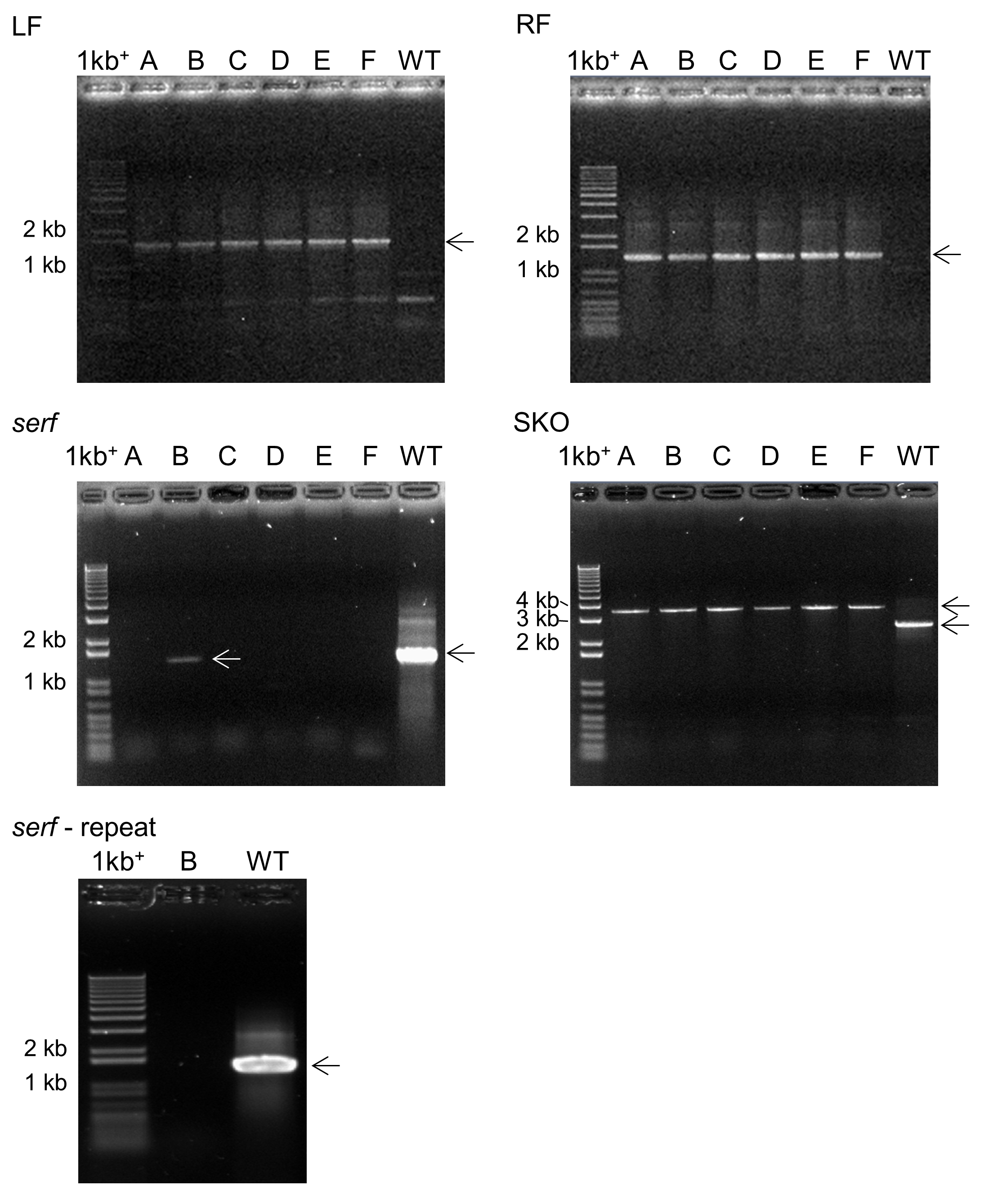

Supplement: Figure S2 — (LF) Left flank of SKO was amplified with primers A/C (Table S2). The 1.6 kb band was present in all mutants (A–F) and absent in the WT. (RF) Right flank of SKO was amplified with primers D/B. The 1.4 kb band was present in all mutants (A–F) and absent in the WT. (serf) serf was amplified with primers SNP1-F/B. The 1.4 kb band was absent in mutants A, C, D, E and F and present in mutant B and in the WT. (SKO) The whole SKO construct was amplified with primers A/B. The 3.6 kb band was present in all mutants and the 2.6 kb band was present in the WT. (serf- repeat) After another purification round for mutant B, no serf DNA could be detected anymore. Arrows indicate the above mentioned bands, WT DNA was used as positive control and size standard was the 1 Kb Plus DNA Ladder™ (Invitrogen). [file peerj-04-2023-s002.png]

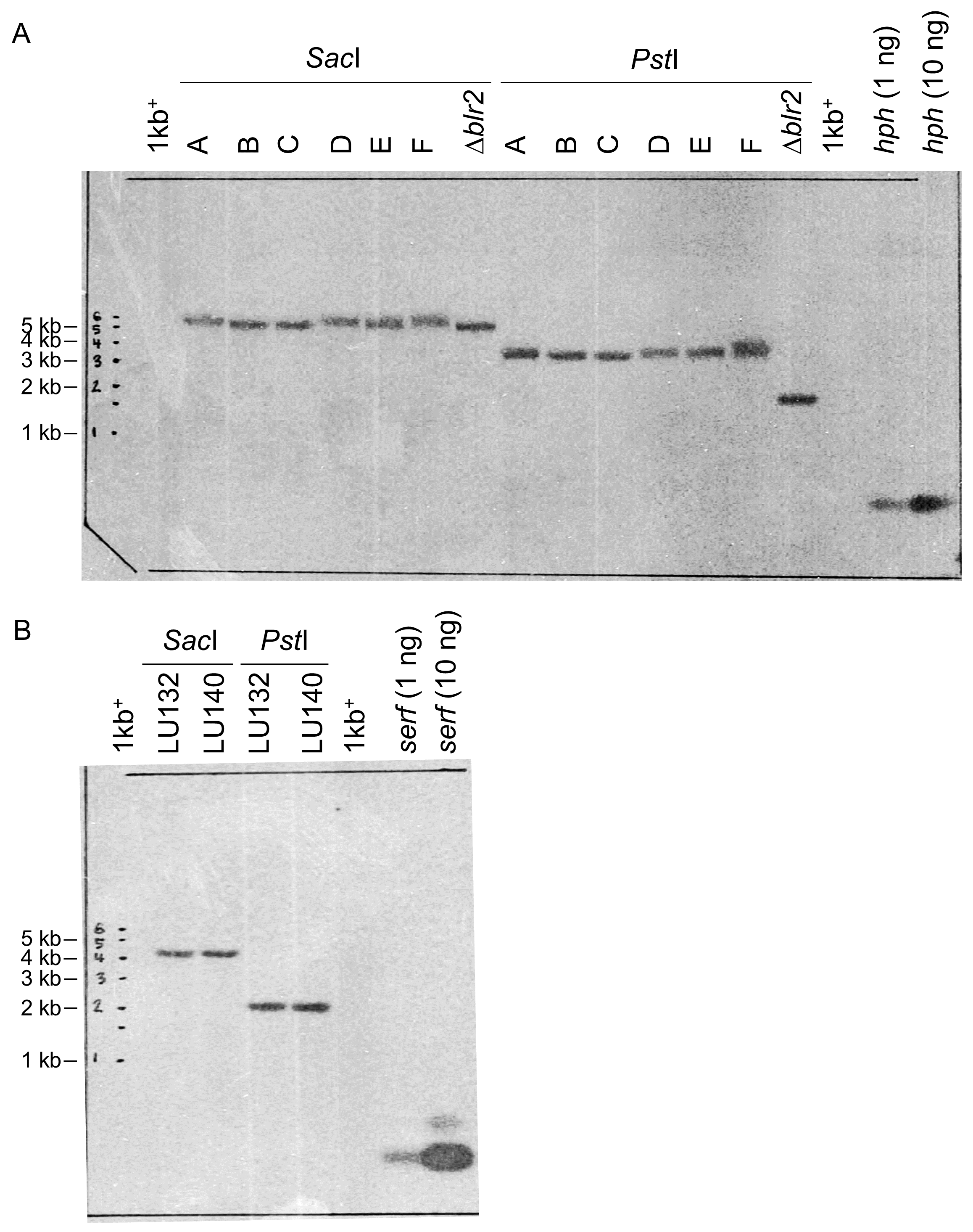

Supplement: Figure 3 — (A) The SacI and PstI digested Δserf mutant DNA was hybridised with the hph probe. A pki/hph containing T. atroviride IMI206040 Δblr-2 mutant was used as positive control. (B) The SacI and PstI digested WT DNA was hybridised with the serf probe. Both hybridisations resulted in single bands, confirming the presence of single copies. Unlabelled probes were used as positive controls and size standard was the 1 Kb Plus DNA Ladder™ (Invitrogen). [file peerj-04-2023-s003.png]

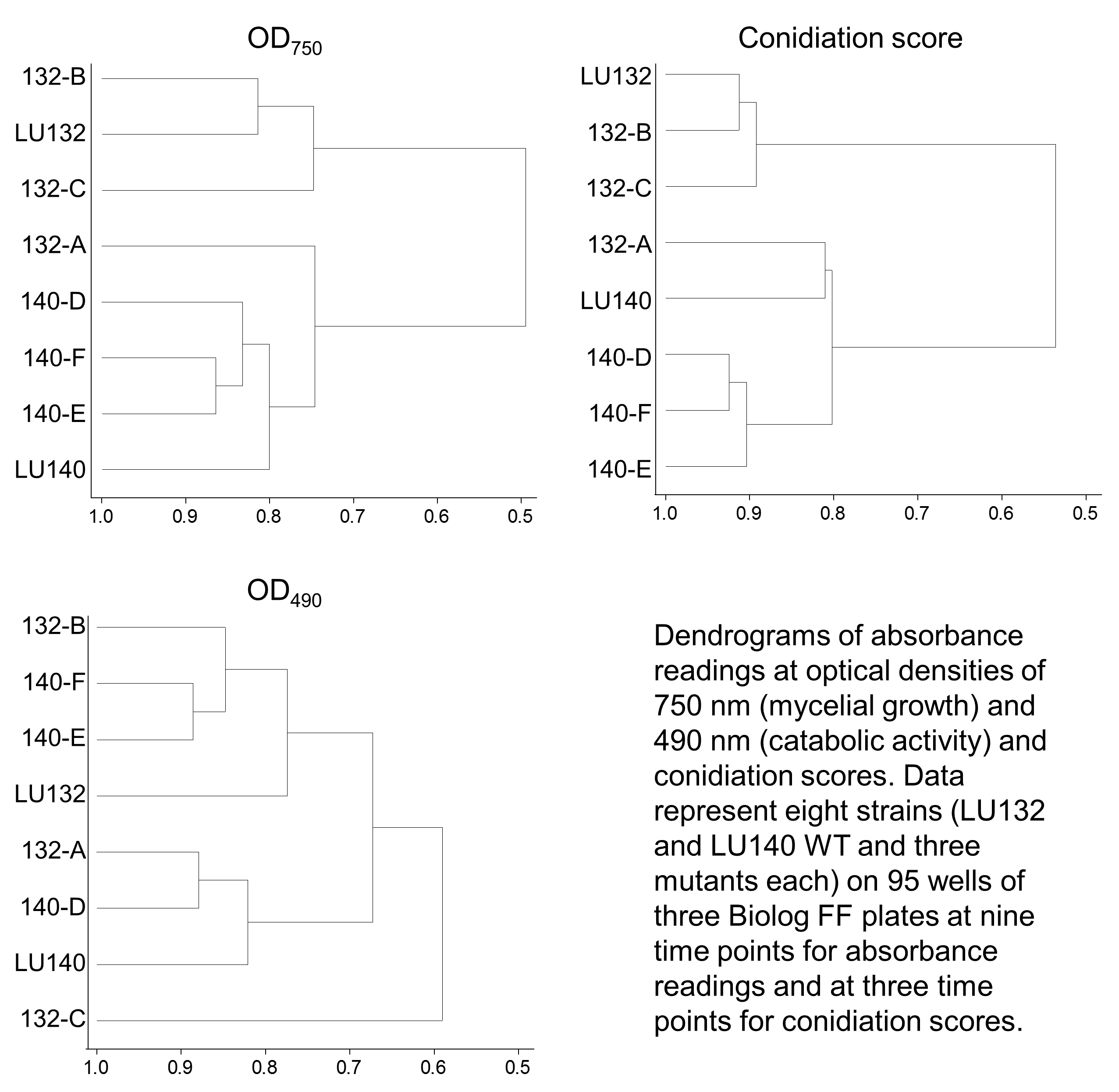

Supplement: Figure S4 — The eight strains were clearly separated into two groups regarding their mycelial growth (OD750) and conidiation on 95 different nutrient sources. The OD490 data were more homogeneous, resulting in higher similarity distances at the branching nodes of the dendrogram. [file peerj-04-2023-s004.png]

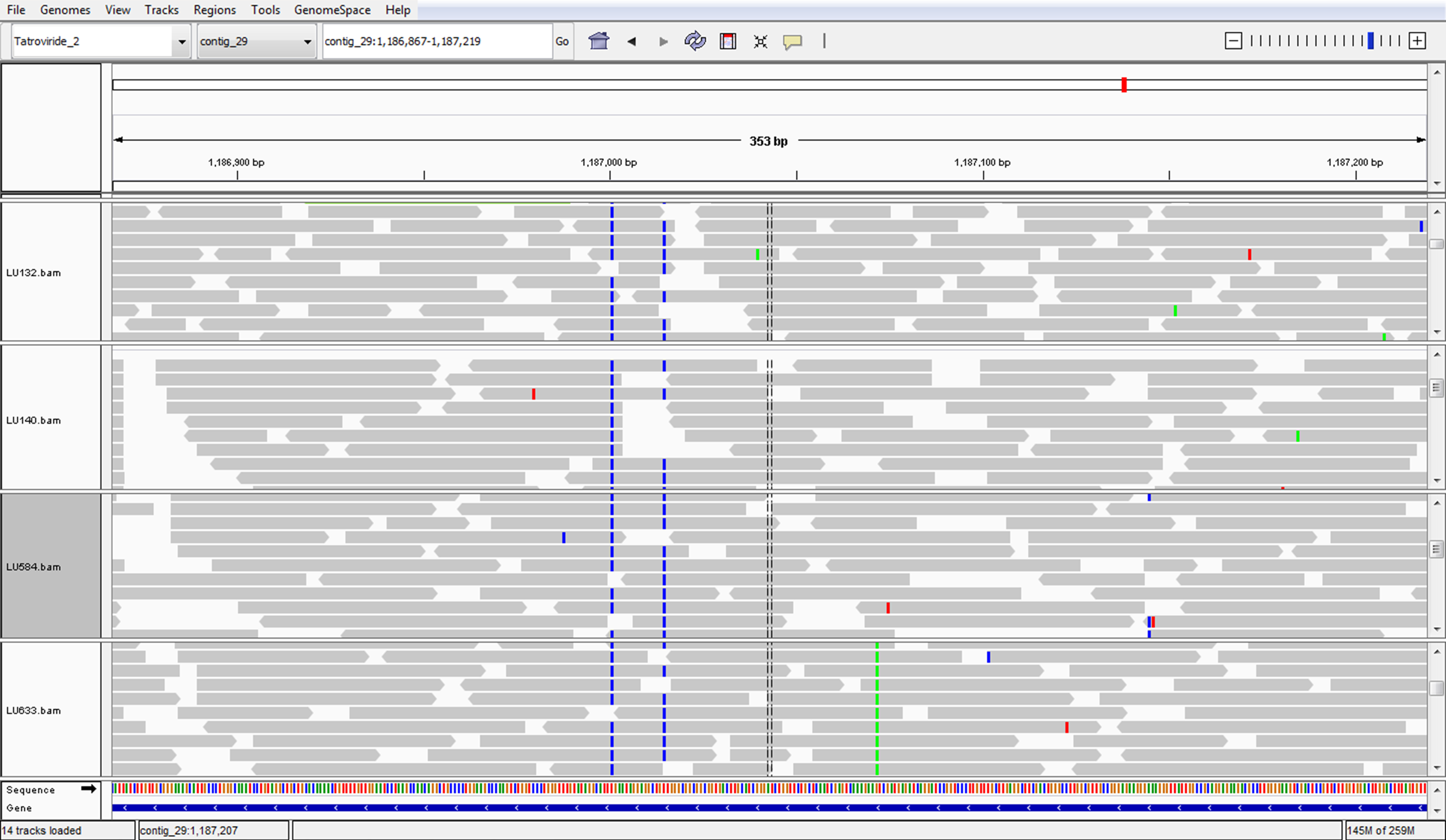

Supplement: Figure S5 — Simple scrolling through the genome sequences enabled the by-chance identification of a SNP (green) in LU633 compared to LU132, LU140, LU584 and T. atroviride IMI206040 (on the bottom panel). [file peerj-04-2023-s005.png]

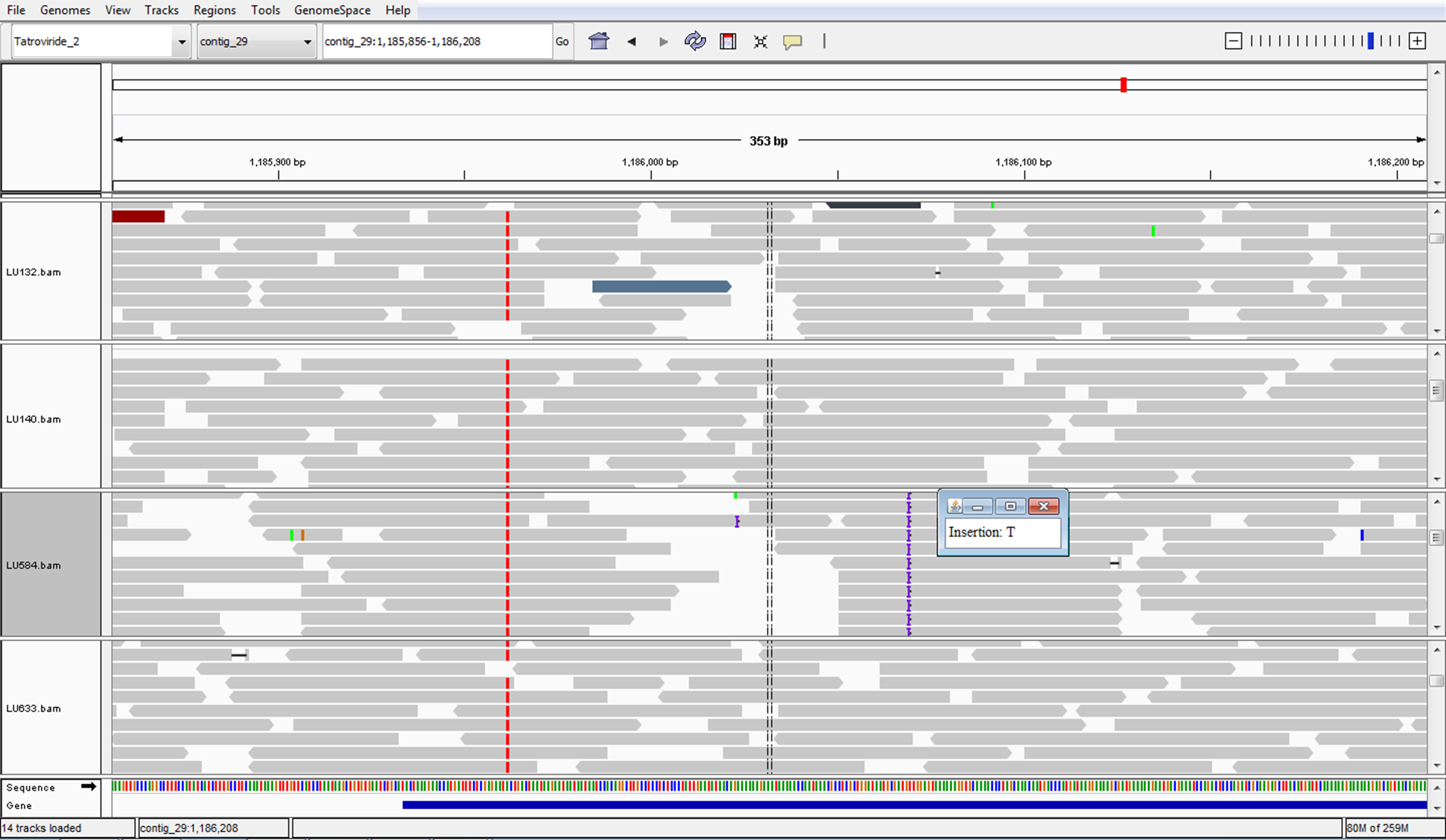

Supplement: Figure S6 — Simple scrolling through the genome sequences enabled the by-chance identification of an insertion (purple) in LU584 compared to LU132, LU140, LU633 and T. atroviride IMI206040 (on the bottom panel). [file peerj-04-2023-s006.png]
